# Supplementary material for: Azithromycin Augments Bacterial Uptake and Anti-Inflammatory Macrophage Polarization in Cystic Fibrosis
Source: Cells. 2024 Jan 16;13(2):166. doi: 10.3390/cells13020166 (PMC10813867; doi:10.3390/cells13020166)
Supplement: Supplementary file 1 [file cells-13-00166-s001.zip › cells-2813010-supplementary.pdf]

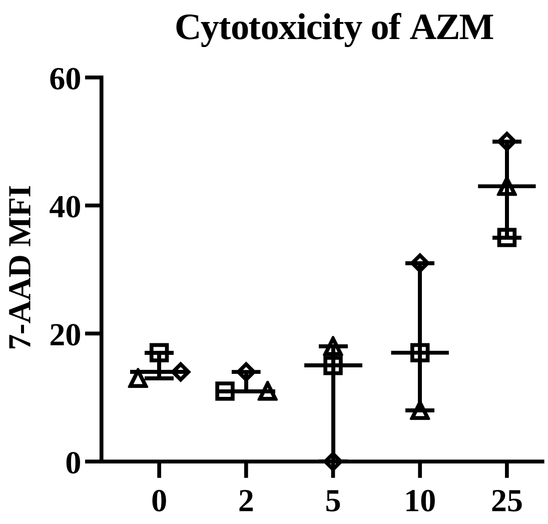

Suppl Fig. S1: Cytotoxicity (7-AAD) of AZM in HC macrophages. AZM at different doses were given during macrophage differentiation. Cytotoxicity of AZM was assessed using 7-AAD DNA binding dye staining by flow cytometry. Each dot represents an independent donor. Data are shown as median (25%, 75%).

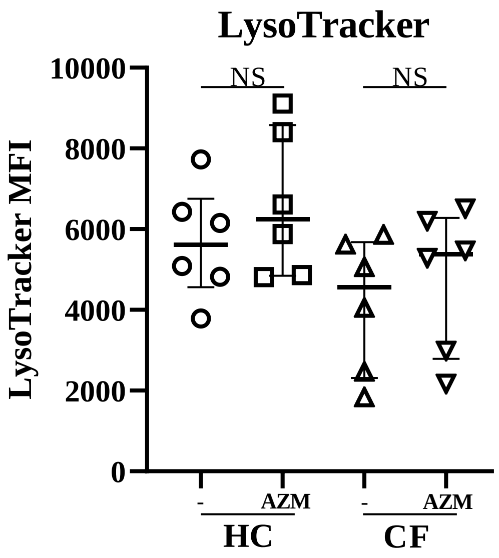

Suppl Fig. S2: Azithromycin has no effect on lysosomal acidification. Lysosomes of PA14 infected cells were stained with LystoTracker green. MFI of Lysotracker was measured by BD Fortessa. Each dot represents an individual healthy donor or pwCF. Data was shown as median (25%, 75%).

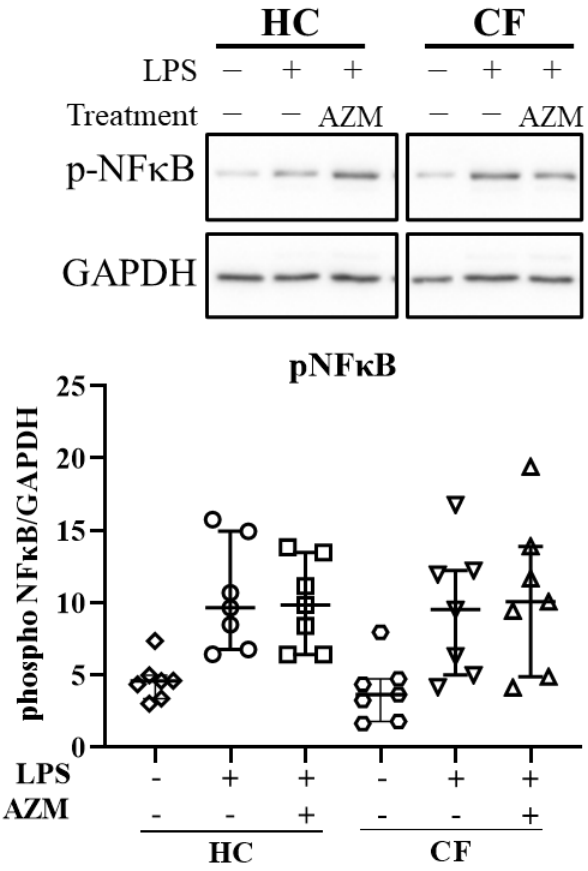

Suppl Fig. S3: Effect of AZM on NFκB activation. MDMs were differentiated in presence or absence of AZM and stimulated with LPS (20ng/ml) for 30min. Phosphorylation of NFκB was analyzed by western blot. Phosphorylation was normalized by the GAPDH level of unstimulated MDMs. Each symbol represents an individual donor or pwCF. Data are shown as median (25%, 75%).
